# Mapping the Binding Landscape of Allosteric Inhibitor G6PDi‑1 on Human G6PD

**DOI:** 10.1021/acs.jpclett.6c01344

**Published:** 2026-06-18

**Authors:** Amit Kumawat, Andrea Perra, Marina Serra, Giorgia Zedda, Marta Anna Kowalik, Andrea Caddeo, Paolo Ruggerone

**Affiliations:** † Department of Physics, 3111 University of Cagliari, Cagliari 09042, Italy; ‡ Department of Biomedical Sciences, Unit of Oncology and Molecular Pathology, University of Cagliari, Cagliari 09042, Italy

## Abstract

Targeting the oxidative pentose phosphate pathway by inhibiting glucose-6-phosphate dehydrogenase (G6PD) is a promising anticancer strategy, yet clinically useful inhibitors remain unavailable. A key limitation is the lack of molecular insight into how allosteric and noncompetitive inhibitors perturb enzymatic activity, limiting rational optimization. Here, we investigated the mechanism of G6PDi-1, a potent, reversible, noncompetitive G6PD inhibitor, by combining biochemical assays with molecular dynamics simulations, Markov state model, and MM/PBSA binding energy calculations. Experimentally, G6PDi-1 reduced active dimer concentration in hepatoblastoma HepG2 cells and increased the inactive monomer fraction, linking inhibition to disrupted oligomerization. Computationally, we identified multiple sites on the enzyme surface, with preferential binding at the dimer interface that sterically blocks oligomerization. Importantly, additional distal sites showed enhanced inter-residue correlations, suggesting secondary allosteric effects that shift G6PD toward monomeric states less competent for oligomerization. These findings provide a molecular basis for structure-based development of improved strategies for G6PD inhibition suited for cancer therapy.

## Graphical abstract

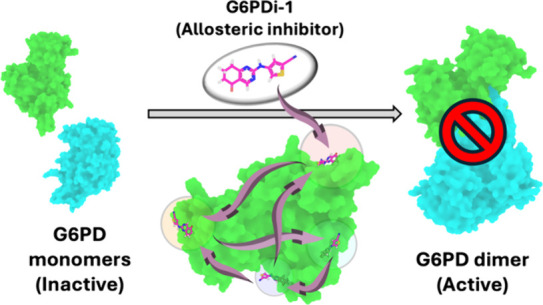

Glucose-6-phosphate dehydrogenase (G6PD, EC 1.1.1.49) is the rate-limiting enzyme of the oxidative pentose phosphate pathway (PPP), which generates ribose-5-phosphate for nucleotide synthesis and NADPH for reductive biosynthesis and antioxidant defense.^1,2^ G6PD expression and activity are upregulated in numerous tumors (e.g., hepatocellular carcinoma (HCC)), contributing to several hallmarks of cancer, including cell proliferation, metastasis, deregulated cellular metabolism and decreased overall survival.^3–9^ Given the key role of the PPP in cancer metabolic reprogramming, it is not surprising that targeting the pathway with specific G6PD inhibitors represents a promising therapeutic option for several cancers, including HCC.^10,11^ Despite this, no G6PD inhibitors have reached clinical use and developing drug-quality inhibitors remains burdensome and challenging. Structurally, human G6PD enzyme exists in an active form as a dimer or tetramer of identical subunits, whereas the monomeric form is catalytically inactive.^12^ Each G6PD monomer contains a substrate binding site for glucose-6-phosphate and two NADP^+^ binding sites, one for catalysis and a second structural site that helps stabilize the enzyme’s oligomeric state.^13,14^

Earlier studies identified steroidal and nonsteroidal small molecules (e.g., dehydroepiandrosterone (DHEA), thienopyrimidines, and benzothiazinones) as putative G6PD inhibitors in several *in vitro* and *in vivo* cancer models, but their clinical translation has been limited by the lack of target specificity and effects that may have resulted from off-target mechanisms rather than direct G6PD inhibition.^15–19^ Rabinowitz and co-workers made a significant breakthrough with the identification of the reversible and noncompetitive inhibitor G6PDi-1 (IC_50_ ≈ 0.07 μM).^20,21^ Although G6PDi-1 is suggested to act allosterically, the molecular mechanism of this effect remains unknown. It has been reported that G6PDi-1 disrupts G6PD oligomerization in human immortalized embryonic kidney HEK293T cells.^22^ We tested G6PDi-1 in human hepatoblastoma HepG2 cells (Cellosaurus RRID: CVCL_0027, DepMap ID: ACH-000739), a cell line widely used for drug safety and toxicity assays, as well as in HCC-related studies, as it displays the key features of a liver neoplastic transformation (see the Supporting Information (SI) for experimental methods).^23,24^ As evident from Figure S1, exposure to G6PDi-1 reduced the dimeric fraction of G6PD and increased the monomeric fraction. This assay, however, does not distinguish whether the reduced dimer population reflects impaired dimer assembly, dissociation of pre-existing dimers, or both. Importantly, this observation extends the effect of G6PDi-1 to a more disease-relevant hepatic cell context and underscores the need to elucidate the molecular basis of this inhibition to support analogue optimization and future inhibitor design.

To investigate the mechanism of G6PDi-1 mediated inhibition, we first used the Schrodinger’s SiteMap tool to identify potential cavities on G6PD based on geometric and physicochemical criteria (Figure S2A, Table S1).^25^ G6PDi-1 was docked into the six identified cavities using Glide tool (Figure S2B),^26^ followed by rescoring of the top poses using MM/GBSA binding free energy calculations (Table S2).^27^ We then performed all-atom molecular dynamics (MD) simulations of the six docked complexes, each representing a distinct binding site to characterize the protein–ligand interaction landscape. For each complex, we ran four independent replicates of approximately 5 μs each, generating a total simulation time of ∼120 μs (see the SI for computational methods). This approach is in line with several studies in which docking is used to generate initial ligand poses, followed by MD simulations to account for ligand–receptor flexibility, and select conformations for structure based virtual screening or mechanistic analysis.^28,29^ Our simulations revealed that G6PDi-1 interacts in a highly dynamic manner exhibiting ligand unbinding events within tens to hundreds of nanoseconds and potential reassociation with the same or alternative regions on the protein surface. We quantified these transient interactions by calculating residue-wise number of contacts between the protein and ligand (Figure 1A). Minimal interactions with the substrate binding site residues corroborate the experimental activity of G6PDi-1 as a noncompetitive inhibitor.^20^ Seven distinct cavities (C1–C7) were defined based on residues exhibiting the highest contact frequency with G6PDi-1, representing localized clusters of frequently contacted surface regions (Figure 1B). C1 and C2 correspond to the region near the catalytic (NADP^+^-C) and structural (NADP^+^-S) cofactor binding sites, respectively; C3 represents the distal pocket located opposite the dimer interface; C4 marks the dimer interface binding region; C5, C6 and C7 denote intermittent sites identified from residues with the highest contact counts. However, the frequent transitions between these sites prevented the identification of a single dominant binding pocket based solely on contact analysis or visual inspection of the trajectories.

**Figure 1:**
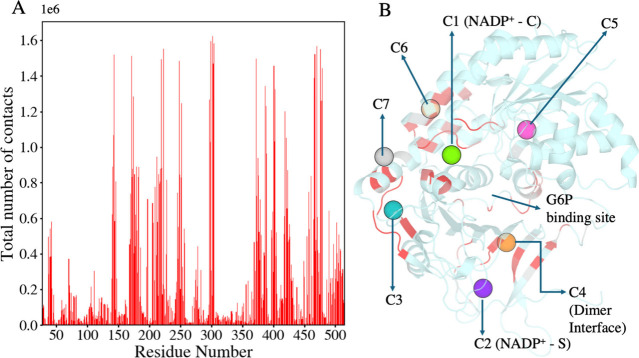
Residue-wise contact analysis and definition of G6PDi-1 binding sites on G6PD. (A) Residue-wise contact plot showing the total number of contacts between G6PDi-1 and G6PD residues observed during the simulations. A contact was defined when the distance between the center of mass (COM) of the ligand and the COM of a residue was less than 0.45 nm. (B) Structural mapping of ligand binding sites on G6PD from the contact analysis. The protein is shown in cartoon representation, with residues exhibiting high contact frequency (>5 × 10^5^ frames) highlighted in red. Seven binding cavities from C1 to C7 were defined based on clusters of residues with high contact frequency.

Subsequently, these extensive transitions between cavities motivated a more quantitative kinetic description using a hidden Markov state model (HMSM) constructed from the MD trajectories (see the SI for more details) using the PyEMMA package.^30–34^ For this purpose, the minimum distance between the COMs of G6PDi-1 and the residues defining each cavity was used as the feature set, allowing an explicit state decomposition of the ligand interaction sites on the G6PD surface. The HMSM was validated using implied timescales (ITS) analysis together with Chapman–Kolmogorov (CK) test. The ITS showed approximate convergence at a lag time of 30 ns, and this lag time was therefore used to construct the final eight-state HMSM. The CK test further showed agreement between predicted and estimated transition probabilities, supporting the statistical robustness of the model (Figure S3A,B).^35^ The resulting kinetic network showed that unbinding from the cavities occurred on relatively similar timescales, with mean first passage times (MFPTs) of approximately 20 μs across multiple sites (Figure 2A). In contrast, association kinetics showed site dependence, with the dimer interface cavity C4 exhibiting the fastest association, with an MFPT of ∼136 μs, followed by C1 and C3, with MFPTs of ∼172 and ∼251 μs, respectively, whereas binding to the structural NADP^+^ site occurred on much slower timescales (∼3 ms), which is likely due to the high flexibility of the C-terminal region shaping this pocket. The ligand bound conformational ensembles therefore represent metastable states sampled on the simulation timescale. We also calculated the stationary populations of the C1–C7 and unbound states from the validated HMSM. The population analysis showed that C3 and C4 are the two dominant ligand bound states, with HMSM-derived populations of approximately 11.7% and 9.8%, respectively, followed by C1 with 7.2%, whereas C2, C5, and C7 were weakly populated (Table S3). We also considered the kinetic accessibility of each cavity using MFPTs from the unbound ensemble. An MFPT cutoff of ∼250 μs was chosen to capture the faster associating sites while excluding slower binding modes in the 0.7–3 ms range. Based on the combination of estimated stationary population and MFPT analysis, the cavities C1 (NADP^+^ catalytic site), C3 (distal pocket), and C4 (dimer interface) were selected as kinetically favored binding regions for further analysis.

**Figure 2:**
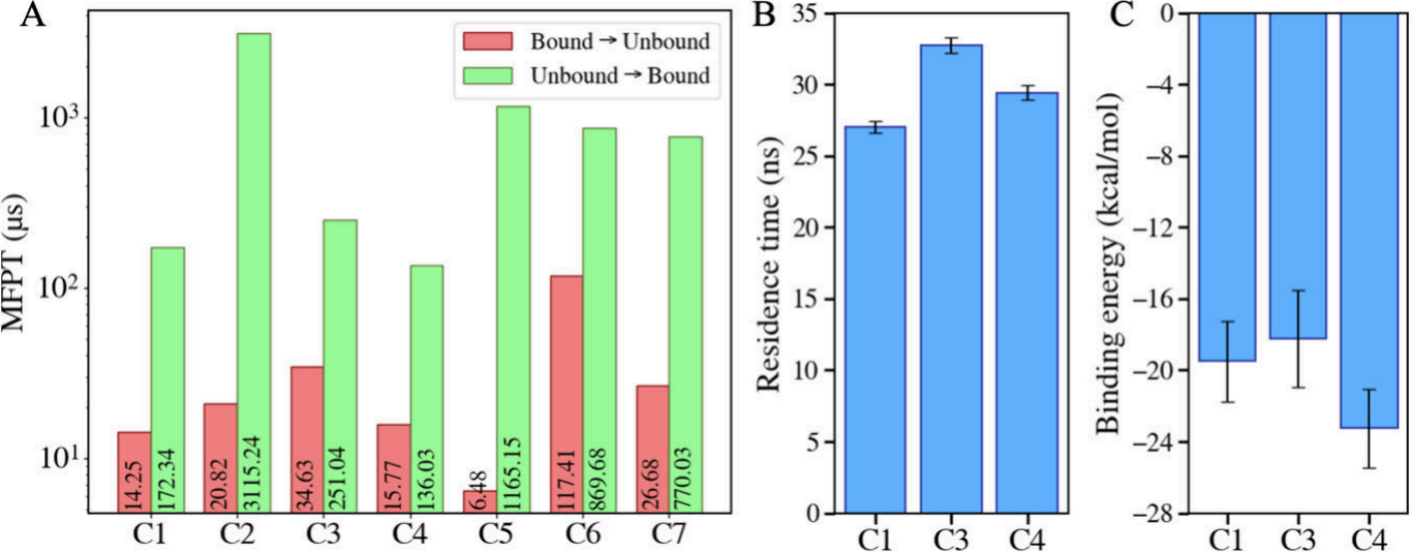
Kinetics and energetics of G6PDi-1 binding to G6PD cavities. (A) MFPTs for ligand transitions between bound and unbound states at each cavity (C1–C7), obtained from the HMSM. Red bars show MFPTs for unbinding (bound to unbound), and green bars show MFPTs for binding (unbound to bound). The logarithmic scale highlights site dependent differences in both association and dissociation kinetics, with fastest binding at the dimer-interface cavity C4 and slowest association at the structural NADP^+^ site C2. (B) Average ligand residence times at the three kinetically favored cavities (C1, C3, C4) derived from MD trajectories, with error bars indicating the standard error of the mean. (C) Binding free energies of G6PDi-1 at cavities C1, C3, and C4 calculated using the MM/PBSA approach on clustered bound ensembles. Error bars represent standard deviations across the sampled frames, showing the most favorable interaction energy at the dimer-interface site C4. All statistical values associated with the kinetic and energetic analyses of cavities C1, C3, and C4 are provided in Table S4.

First, the average residence time of G6PDi-1 in these three cavities (C1, C3, C4) was determined over all trajectories. A ligand was considered bound when the distance between the COMs of G6PDi-1 and the corresponding cavity residues is less than 0.5 nm. We observed multiple binding and unbinding events, and residence time distributions were fitted with a single exponential function to obtain the average residence time (τ) for each site (Figure 2B). G6PDi-1 remains bound for ∼27 and ∼29 ns at the catalytic NADP^+^ site (C1) and the dimer interface (C4), respectively, whereas the distal pocket (C3) exhibited a slightly extended occupancy of ∼33 ns. Ligand bound frames corresponding to these sites were then extracted and clustered to analyze the ligand conformations at each pocket. Representative structures from the most populated clusters showed that G6PDi-1 adopts heterogeneous conformations at C1 and C4, consistent with local protein flexibility and the solvent exposed nature of these regions (Figure S4). In contrast, binding at C3 was associated with a narrower distribution of ligand poses, indicative of a more constricted local environment.

It is worth emphasizing that the kinetic parameters reported here should not be interpreted as direct predictors of the experimentally measured inhibitory potency (IC50). The reported MFPTs and residence times describe ligand binding events between G6PDi-1 and individual cavities of the monomeric protein under near-equilibrium simulation conditions. In contrast, the IC50 value reflects a macroscopic functional response of the enzyme that is not explicitly captured by the monomeric binding model, including redistribution of the conformational ensemble, reduced formation of active oligomeric species, and repeated transient encounters with several allosteric regions.^36,37^ Thus, functional inhibition by a reversible, noncompetitive inhibitor such as G6PDi-1 may not arise from tightly bound occupancy at a single pocket; instead, frequent transient interactions across kinetically accessible sites may be sufficient to shift the population between inactive monomeric and active oligomeric states.^38–41^ The kinetic analysis is therefore used to identify and compare accessible binding regions that may contribute to inhibition, rather than to derive a quantitative relationship between residence time and IC50.

Binding free energy calculations were subsequently performed using the MM/PBSA approach for the frames belonging to the top clusters that cumulatively accounted for 75% of the bound ensemble at each site.^42,43^ Despite the higher ligand fluctuations and shorter residence time, the dimer interface pocket (C4) has more favorable binding energetics for G6PDi-1, followed by C1, whereas the distal pocket (C3) shows comparatively weaker interaction energy among the three sites (Figure 2C). This difference between residence time and energy-based ranking highlights that the residence time analysis captures transient ligand occupancy, whereas binding energy calculations estimate the relative energetic stabilization of selected clustered bound conformations. Furthermore, decomposition of the binding free energies into residue-wise contributions showed that hydrophobic contacts and intermittent hydrogen bonds (H-bonds) stabilize the ligand at all three sites. (Figure S5). For example, at C1, both polar and nonpolar interactions contribute to ligand stabilization, including H-bonds with Tyr249 and Lys171 (Figure S5B). The distal pocket (C3) is characterized by a predominantly hydrophobic enclosure formed by aliphatic residues (e.g., V303, Leu468, Pro477, I480) that restrict ligand motion, together with consistent H-bond with Leu305 backbone (Figure S5D). Similarly, at the highest affinity dimer interface site (C4) in Figure S5F, binding is driven by hydrophobic interactions (e.g., Ile220, Phe373, Leu390, Met404, Leu420) combined with H-bonds involving residues such as Asn218, Asn388, Asp389, Thr402 and Ser418, which together result in the most favorable overall binding energy. This finding supports the experimental evidence of disrupted oligomerization, providing a rationale for observed inhibition.

In order to investigate the plausible allosteric modulation through ligand binding at these sites, we compared the conformational dynamics of the ligand bound states (C1, C3, and C4) with the MD simulation trajectories (three replicates, 100 ns each) for the active tetrameric state containing substrate G6P and NADP^+^ at the catalytic and structural sites. Additionally, we performed MD simulations of *apo* monomeric G6PD in the absence of ligand (four replicates, 1 μs each) to assess the flexibility of the interface-forming region and to identify the structural features that stabilize the dimer interface in the oligomeric state. The active G6PD form showed a narrower interface RMSD distribution than the monomeric state, together with a stable intersubunit contact area (Figure S6A,B). Furthermore, native contact analysis identified intersubunit salt bridges, including D228–R219, D375–R219, E206–K407 and E419–R427, that stabilize the interface (Figure S6C). These residues overlap with regions showing enhanced flexibility in the monomeric state (Figure S6D), indicating that oligomerization stabilizes the interface through specific intersubunit interactions that are absent or weakened in the *apo* monomer. Similarly, Figure S7 shows an increase in root-mean-square fluctuations (RMSF) in the G6PDi-1 bound systems within residues forming inter-subunit contacts and the dimer interface due to the disrupted intersubunit packing as compared to the active form. We therefore next asked whether ligand binding also allosterically alters internal communication,^44–46^ and to address this we computed residue-wise normalized linear mutual information (nLMI) for the active and ligand bound systems (Figure S8).

Figure 3A–C shows the difference in the nLMI for these systems, calculated as Δ*nLMI* = *nLMI*_*Cavity*_ – *nLMI*_*Active*_, where positive values (blue) indicate stronger inter-residue coupling in the ligand bound state and negative values (red) indicate stronger coupling in the active state. The nLMI matrices for ligand bound at cavities C1 and C3 show broader off-diagonal coupling than the active state (Figure S8B,C), and their differential maps are dominated by positive values (Figure 3A,B), indicating increased inter-residue coupling upon ligand binding. In contrast, the ligand bound C4 state shows a near neutral profile with only limited and localized changes (Figures S8A,D and 3C), consistent with a direct mechanism linked to the interface occlusion. This hypothesis was further supported by quantifying the population distribution of conformations retaining a dimer-like interface during the simulations. Since the HMSM was constructed using the distance between G6PDi-1 and plausible binding sites, it resolves ligand bound states on the monomer surface but does not directly distinguish oligomeric states. Therefore, we compared the intrinsic conformational distributions of the dimer-like interface in the monomer (*apo*) simulations with the ligand bound ensembles. For this purpose, the active state trajectories were clustered using residues forming the dimer interface, and the representative structure from the most populated cluster was used as the reference for dimer-like conformation. We then calculated distance RMSD (DRMSD) for the interface forming residues using PLUMED package for the monomer (*apo*) trajectories and for the ligand bound C1, C3 and C4 ensembles. The *apo* monomer sampled dimer-like interface conformations in approximately 27% of frames (Figure 3D), whereas this population decreased to 4–7% in the ligand bound states, supporting the proposed mechanism that G6PDi-1 reduces the population of dimer-competent monomeric conformations. Structural mapping of the selective highly correlated long-range nLMI residue pairs further suggested plausible allosteric pathways linking ligand interacting residues at C1 and C3 to the dimer interface (A217, N218, F381, V400, D421, and Y424), indicating that perturbations at these sites may propagate toward regions involved in dimer-interface packing (Figure S9). Thus, our results support a distinction between the sites, where ligand binding at the C4 site directly interferes with oligomerization, whereas ligand binding at distal sites C1 and C3 are associated with enhanced inter-residue correlations that shift the conformational ensemble away from dimer-competent states.^47,48^

**Figure 3:**
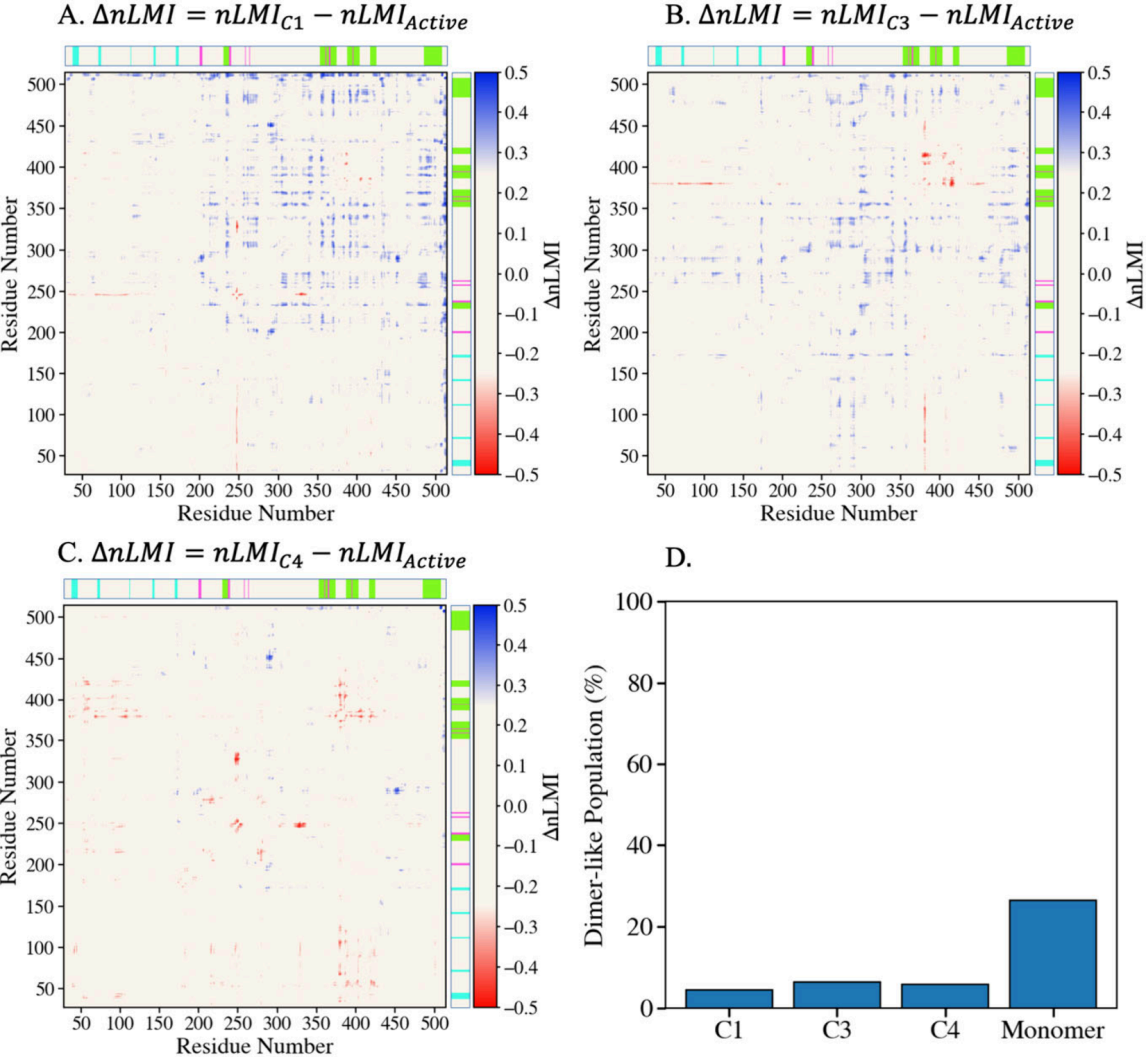
Ligand induced changes in residue–residue coupling and dimer-like interface conformations in G6PD. (A–C) Differential residue-wise Δ*nLMI* maps comparing ligand bound states with the active form. The annotated colored dashes along the top and right sides of each Δ*nLMI* map indicate residues associated with the substrate-binding site (magenta), structural NADP^+^ site (green), and catalytic NADP^+^ site (cyan). Ligand binding at cavities C1 (A) and C3 (B) indicates enhanced inter-residue correlations relative to the active state. In contrast, the C4-bound state (C) shows only localized changes with a similar profile to the active state. (D) Population (%) of dimer-like interface conformations in *apo* and G6PDi-1 bound ensembles. The *apo* monomeric G6PD ensemble sampled dimer-like interface conformations more frequently, whereas G6PDi-1 bound states at C1, C3, and C4 showed a reduced dimer-like population.

We must note that these findings provide a mechanistic description of ligand-accessible states on monomeric G6PD, rather than a complete thermodynamic model of G6PDi-1-dependent redistribution of oligomeric states, and therefore, several methodological constraints should be considered when interpreting the results. The use of a monomeric form, though, allowed extensive sampling of G6PDi-1 across the protein surface and identification of kinetically and energetically preferred interaction regions. However, it cannot directly quantify the ligand-induced shifts in the monomer–oligomer equilibrium in cells and, in turn, does not address whether G6PDi-1 can bind and destabilize a pre-existing G6PD dimer. In addition, residence times and MFPTs should be interpreted as comparative descriptors, because they were derived from distance-based definitions of bound states and simplified fitting procedures, and slower events may not be quantitatively resolved within the accessible simulation timescales. Moreover, end-point energy approaches such as MM/PBSA and MM/GBSA are sensitive to the conformational ensemble selected for the calculation and do not explicitly include entropic contributions associated with ligand diffusion, large-scale conformational changes, or oligomerization equilibria.

In summary, we combined biochemical data with MD simulations and binding free energy calculations to map the binding landscape of the allosteric G6PD inhibitor G6PDi-1 and define its likely mechanism of action. We demonstrate that G6PDi-1 reduces the active dimeric form in HepG2 cells and this phenotype is supported computationally by preferential ligand interaction with the dimer-interface region, increased flexibility of interface-forming residues, and a reduced population of dimer-compatible monomeric conformations. Interestingly, G6PDi-1 binding at distal sites reshapes the inter-residue coupling network, uncovering an additional mode of allosteric modulation. Together, these findings unearth the structural and mechanistic basis of G6PDi-1 inhibition and establish a foundation for structure-based development of new G6PD inhibitors for effective antitumoral therapy.

## Supplementary Material



## Data Availability

The MD simulation data sets that support the conclusions of this article are available in the Zenodo repository with the following doi: 10.5281/zenodo.20305884.

## Notes

Experimental procedures, computational methods, Supplementary Tables S1–S4, and Supplementary Figures S1–S12 (PDF)
