# Supplementary material for: Mapping the Binding Landscape of Allosteric Inhibitor G6PDi‑1 on Human G6PD
Source: J Phys Chem Lett. 2026 Jun 18;17(26):7267–73. doi: 10.1021/acs.jpclett.6c01344 (PMC13339762; doi:10.1021/acs.jpclett.6c01344)
Supplement: Supplementary file 1 [file jz6c01344_si_001.pdf]

Mapping Binding Landscape of Allosteric Inhibitor G6PDi-1 on Human G6PD

Amit Kumawat,[1,*] *Andrea Perra,*[2] *Marina Serra,*[2] *Giorgia Zedda,*[2] *Marta Anna Kowalik,*[2] *Andrea Marco Caddeo,*[2] *Paolo Ruggerone*[1]

[1] Department of Physics, University of Cagliari, Italy

[2] Department of Biomedical Sciences, Unit of Oncology and Molecular Pathology, University of Cagliari, Italy

Corresponding Author

* Amit Kumawat: amit.kumawat@unica.it

The supporting information includes:

Materials and methods

1. **Experimental details:**
    - (a) Cell cultures and G6PDi-1 treatment
    - (b) Protein extraction and sample preparation
    - (c) Western blotting analysis
2. **Computational details:**
    - (a) Binding site prediction and molecular docking protocol
    - (b) G6PDi-1 molecule parameterization
    - (c) Molecular dynamics simulations of ligand bound G6PD
    - (d) Molecular dynamics simulations of wild type tetrameric G6PD
    - (e) Molecular dynamics simulations of wild type single subunit monomeric G6PD
    - (f) MSM estimation and validation

Supplementary tables (S1 to S4) with legends:

Table S1. Scoring of G6PD binding pockets identified by SiteMap analysis.

Table S2. Docking scores and MM-GBSA scoring for the best docked poses of G6PDi-1.

Table S3. Stationary populations estimated from the HMSM.

Table S4. Statistical summary of kinetic and energetic analyses for G6PDi-1.

Supplementary figures (S1 to S12) with legends:

S1. Effect of G6PDi-1 on dimer formation in hepatoblastoma HepG2 cells.

S2. Identified binding pockets and top docked poses of G6PDi-1 on G6PD.

S3. Hidden Markov state model (HMSM) of G6PDi-1 cavity exchange on G6PD.

S4. Representative conformations of G6PDi-1 at the binding cavities (C1, C3 and C4).

S5. Residue-wise decomposition of the MM/PBSA binding free energy at C1, C3, and C4.

S6. Structural stability analysis for the interface forming region.

S7. RMSF between the ligand bound systems (C1, C3, C4) and the active tetrameric state.

S8. Pairwise nLMI matrices for the active and G6PDi-1 bound at cavities C1, C3 and C4.

S9. Allosteric pathways between ligand interacting regions and the dimer interface.

S10. RMSD plot of G6PD monomer for the six docked protein-ligand systems.

S11. RMSD plot of G6PD tetramer in active state.

S12. RMSD distribution violin plot for all systems.

References

Materials and methods

1. Experimental details

(a) Cell cultures and G6PDi-1 treatment

HepG2 (ATCC-HB-8065) cells were grown in DMEM high-glucose (Euroclone, #ECB7501L, Lot n°EUM01HO) supplemented with 10% fetal bovine serum (FBS) (Euroclone, ECS-5000L, Lot n° EUS53040524), 1% glutamine (Thermo Fisher Scientific, 35050061, Lot n° 3224711), 1% of MEM Non-essential amino acids (SIAL-NEAA-B, Lot n° 23-6365) and 1% penicillin/streptomycin (Euroclone, #ECB3001D, Lot n° EUM01O9). HepG2 cells (2x10^6 cells/flask) were seeded into 75 cm^2 flask. The day after cells were treated with 100 µM of G6PDi-1 (HY-W107464, MedChemExpress) for 24 hours, while control cells were treated with the same amount of dimethyl sulfoxide (DMSO).

(b) Protein extraction

G6PDi-1 treated and control HepG2 cells were washed with phosphate-buffered saline (PBS) and harvested by trypsinization for 5 min at 37°C. Trypsin activity was neutralized with complete culture medium. Cells were then incubated on ice for 1 hour with Cell Lysis Buffer (1X) (Cell signalling #98039), used to lyse cells under nondenaturing conditions, supplemented with Halt protease and phosphatase inhibitors (Thermo Fisher Scientific, #78444) and 0,9% of Triton X-100 (Sigma-Aldrich #282103). The lysate was centrifuged at 12000 rpm at +4°C for 15 minutes and the supernatant was recovered. Protein concentrations were measured by spectrophotometer.

(c) Western blotting analysis

60 ug of cell lysate were loaded in Criterion TGX Stain-Free 10% Precast gel (Biorad, #5671034) and then transferred onto nitrocellulose membrane at 300mA for 90 minutes. The membranes were then incubated in non-fat dry milk 5%, Tween-20 0,05% in TBS at room temperature for 1 hour. The immunoblot was probed with primary and appropriate secondary

antibodies against G6PD (Abcam, ab87230). Vinculin was used as endogenous control (Abcam, ab129002). Monomeric and dimeric G6PD bands were detected using a chemiluminescence detection system with the Invitrogen iBright Imaging System (Thermo Fisher Scientific).

2. Computational details

(a) Binding site prediction and molecular docking protocol

Potential binding site prediction and molecular docking were carried out using the Schrödinger Suite (Schrödinger, 2024) SiteMap module and the molecular modeling package.[1,2] A monomer structure from the tetrameric crystal structure of human G6PD (PDB ID: 7SNI)[3] was used as the template. The wild type structure was obtained by back replacing residue Asp200 to Asn. Protein preparation was performed with the Protein Preparation Wizard. Hydrogen atoms were added, hydrogen bonding networks optimized, and the structure subjected to restrained energy minimization. Binding sites on G6PD were predicted and characterized using SiteMap with default parameters. The top six predicted sites (**Figure S2A and Table S1**) were selected for grid generation. Residues within a 5 Å radius of the binding site were set as flexible. Docking calculations were performed using the Glide SP scoring function,[4] followed by MM-GBSA binding free energy calculations with the OPLS4 force field and the VSGB 2.1 solvation model.[5,6] The top-ranked docking pose for each cavity was selected based on the docking score and Glide gscore and MM-GBSA binding energy[7] (**Figure S2B and Table S2**) for subsequent molecular dynamics (MD) simulations.

(b) G6PDi-1 molecule parameterization

Parameters of the G6PDi-1 ligand for the MD simulations were generated using the AmberTools24 package in combination with Gaussian16.[8–10] The initial 3D structure of the ligand was converted into a GAFF2 compatible mol2 file and assigned AM1-BCC charges with Antechamber.[11] To obtain accurate atomic charges for MD simulations, a three-step quantum

mechanical protocol was employed using Gaussian16 package.[8] First, the ligand geometry was optimized at the Hartree-Fock/PM7 level with tight SCF convergence.[12] This was followed by a refined geometry optimisation at the B3LYP/def2-SVP level of theory including Grimme's D3 dispersion correction and an ultrafine integration grid to ensure a stable minimum.[13–15] Finally, single point electrostatic potential calculations were performed at the HF/6-31G* level using the Merz-Kollman scheme, which provided the input for restrained electrostatic potential (RESP) charge fitting.[16,17] The RESP derived charges were subsequently assigned to the ligand atoms using Antechamber with the GAFF2 force field.[11] Any missing bonded or torsional parameters were identified with the parmchk2 utility and compiled into a forcefield modification file. The final outputs, consisting of a GAFF2 mol2 topology and an frcmod parameter file, were then converted into Gromacs format using ParmEd package.[18]

(c) Molecular dynamics simulations for G6PDi-1 bound complexes

MD simulations were performed for G6PD in complex with G6PDi-1 docked at six distinct binding sites. For each system (docked complex), four independent replicas were simulated for approximately 5.0 μs each using a 2 fs integration time step. All simulations were performed using GROMACS 2024.2[19] with the Amber19SB force field[20] to model the protein, and the TIP3P water model.[21] Each system was solvated in a periodic box of water molecules and neutralized by the addition of counterions. To mimic physiological conditions, Na^+ and Cl^- ions were added to reach a final salt concentration of 150 mM. Neighbor searching was performed every 20 steps. The PME algorithm was used for electrostatic interactions with a cutoff of 0.9 nm.[22] A reciprocal grid of 100 x 80 x 96 cells was used with 4th order B-spline interpolation. A single cutoff of 0.93 nm was used for Van der Waals interactions. All bond lengths involving hydrogen atoms were constrained using the LINCS algorithm.[23] The temperature was maintained at 310 K using the V-rescale thermostat,[24] and pressure was maintained at 1 atm using the C-rescale barostat,[25] both under periodic boundary conditions.

All systems were energy minimized using the steepest descent algorithm, followed by a multistep equilibration protocol. For each replica, frames were saved every 10 ps. The total production time generated from these simulations is around 120 µs. All systems were found to be stable during the simulations (**Figure S10**).

(d) MD simulations for the wild type G6PD in active tetrameric form

The cryo-EM structure of tetrameric G6PD bound to NADP+ and glucose-6-phosphate (G6P) (PDB ID: 7SNI)[3] was used to generate the wild type structure. To generate the wild type model, residue 200 was back mutated from Asp to Asn. All simulations were carried out using GROMACS package[19] with the CHARMM36 force field[26] and the TIP3P water model.[21] The parameters for G6P and NADP+ were generated using CGenFF.[27] Each system was neutralized, followed by energy minimization with the steepest descent algorithm. The system was equilibrated with temperature maintained at 300 K using the V-rescale method[24] and pressure kept constant at 1 atm using the C-rescale barostat.[25] Neighbor searching was performed every 10 steps. A reciprocal grid of 96 x 96 x 96 cells was used with 4th order B-spline interpolation. All simulations were performed using periodic boundary conditions, and the particle mesh Ewald method was used for the calculation of electrostatic interactions.[22] The cut-off distance for electrostatic and van der Waals interactions was set to 1.0 nm. Three independent simulations were run with a time step of 2 fs for 100 ns each, with frames saved at every 10 ps. All trajectories corresponding to the active tetramer state were found to be stable during the simulations (**Figure S11**).

(e) MD simulations for the wild type G6PD in monomeric form in the absence of ligand

The cryo-EM structure of tetrameric G6PD bound to NADP+ and glucose-6-phosphate (G6P) (PDB ID: 7SNI)[3] was used to generate the wild type single subunit monomeric (*apo*) structure in the absence of any ligand. To generate the wild type model, residue 200 was back mutated from Asp to Asn. All simulations were performed using GROMACS 2024.2[19] with the

Amber19SB force field[20] to model the protein, and the TIP3P water model.[21] To mimic physiological conditions, Na^+ and Cl^- ions were added to reach a final salt concentration of 150 mM. Neighbor searching was performed every 20 steps. The PME algorithm was used for electrostatic interactions with a cutoff of 0.9 nm.[22] A reciprocal grid of 96 x 96 x 96 cells was used with 4th order B-spline interpolation. A single cutoff of 0.93 nm was used for Van der Waals interactions. Each system was neutralized by the addition of counterions, followed by energy minimization with the steepest descent algorithm. The system was equilibrated with temperature maintained at 310 K using the V-rescale method[24] and pressure kept constant at 1 atm using the C-rescale barostat.[25] Four independent simulations were run with a time step of 2 fs for 1000 ns each, with frames saved at every 10 ps. The RMSD distribution plot show that all trajectories corresponding to the *apo* monomeric G6PD state were found to be stable during the simulations (**Figure S12**).

(f) MSM estimation and validation

We built a hidden Markov state model (HMSM) to describe how G6PDi-1 transients between multiple cavities on G6PD using PyEMMA.[28,29] All MD trajectories from the six docked complexes (four replicas each) were used for MSM construction. Seven binding cavities (C1-C7) were defined from contact analysis, and for every frame we computed the minimum distance between the COM of ligand and the COM of residues used to define each cavity. Frames were assigned to a given cavity if the distance was within a cavity-specific cutoff ranging from 0.4-0.6 nm; otherwise, frames were labeled unbound, giving eight observable states in total. The resulting discrete trajectories were passed to PyEMMA to estimate an eight-state HMSM at a lag time of 30 ns. The lag time was selected from implied timescale analysis and the model was further validated by a Chapman-Kolmogorov test (**Figure S3A, B**). The validated HMSM was then used to derive stationary populations and kinetic observables for ligand exchange between bound cavities and the unbound ensemble (**Figure S3C**).

Supplementary tables (S1 to S4)

Table S1. Scoring of G6PD binding pockets identified by SiteMap analysis using Maestro.

Title	SiteScore	Exposure	Philic	Phobic	Volume (Å^3)	Residue Number
Site 1	0.97	0.74	0.40	0.60	206.49	58,60,206,209,210,212,213,216,217,275,276,278,279,424,432,434,438,439,442,443,448,450,451
Site 2	0.97	0.59	0.75	0.63	177.12	175,251,252,253,254,256,257,329,332,333,334,335,469,472,473,476
Site 3	0.97	0.74	0.35	0.57	295.45	206,210,213,214,218,220,221,224,228,229,373,374,375,376,377,381,388,400,401,402,404,405,406,407,415,420,422,424,428,431,433
Site 4	0.96	0.62	0.65	0.33	833.02	38,40,41,42,43,46,47,54,57,72,73,87,88,89,112,141,142,143,144,145,146,148,170,171,172,174,175,179,182,183,201,202,205,206,237,239,241,243,244,246,249,250,252,253,258,259,263,360,365,395,396,397,398,425,431,432,433,434,435,436,437
Site 5	0.95	0.76	0.39	0.51	118.55	300,301,303,304,305,468,471,472,475,477,478,479,480,494,495,498
Site 6	0.93	0.67	0.68	0.25	627.26	236,238,357,363,364,365,366,368,370,372,386,387,389,393,396,397,401,403,406,409,416,417,418,419,421,422,423,426,427,429,487,493,494,496,497,501,502,503,504,505,506,507,508,509,510,511,512,513,515

Table S2. Docking scores and MM-GBSA scoring for the best docked pose of G6PDi-1 in each SiteMap identified pocket. The table is organized with docking poses ranked by most favorable MM-GBSA binding energy ($kcal/mol$).

Docking Index	Site Name	Docking score	Glide gscore	ΔG $(kcal/mol)$
Dock-1	Site-2	-5.19	-5.19	-45.20
Dock-2	Site-3	-5.12	-5.12	-43.13
Dock-3	Site-6	-4.13	-4.13	-38.66
Dock-4	Site-4	-4.40	-4.40	-37.66
Dock-5	Site-1	-5.06	-5.06	-36.34
Dock-6	Site-5	-4.92	-4.92	-35.44

Table S3. Stationary populations of G6PDi-1 across G6PD binding sites estimated from the HMSM

State	Stationary population (%)
C1	7.20
C2	0.65
C3	11.73
C4	9.83
C5	0.60
C6	6.03
C7	1.79
Unbound	62.18

Table S4. Statistical summary of kinetic and energetic analyses for G6PDi-1 binding at cavities C1, C3, and C4

Cavity	MFPT (μs)		Residence time (ns)	Binding energy (kcal/mol)
	Unbound to Bound	Bound to Unbound		
C1	172.3 ± 83.16	14.24 ± 3.55	27.03 ± 0.39	-19.51 ± 2.27
C3	251.03 ± 113.23	34.6 ± 11.31	32.71 ± 0.54	-18.23 ± 2.73
C4	136.03 ± 43.69	15.76 ± 4.01	29.43 ± 0.48	-23.26 ± 2.19

Supplementary figures (S1 to S11)

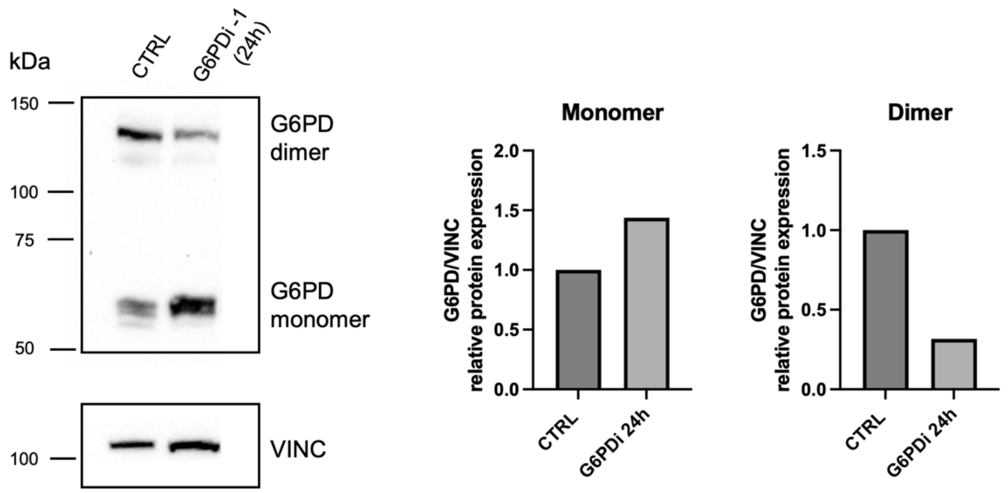

Figure S1. G6PDi-1 blocks formation of G6PD dimer in hepatoblastoma HepG2 cells. HepG2 cells were treated with G6PDi-1 at the concentration of 100 μM for 24 hours. Control cells (CTRL) were treated with DMSO. Western Blot of G6PD protein levels. Vinculin (VINC) was used as loading control. Western blot quantification was calculated using ImageJ software.

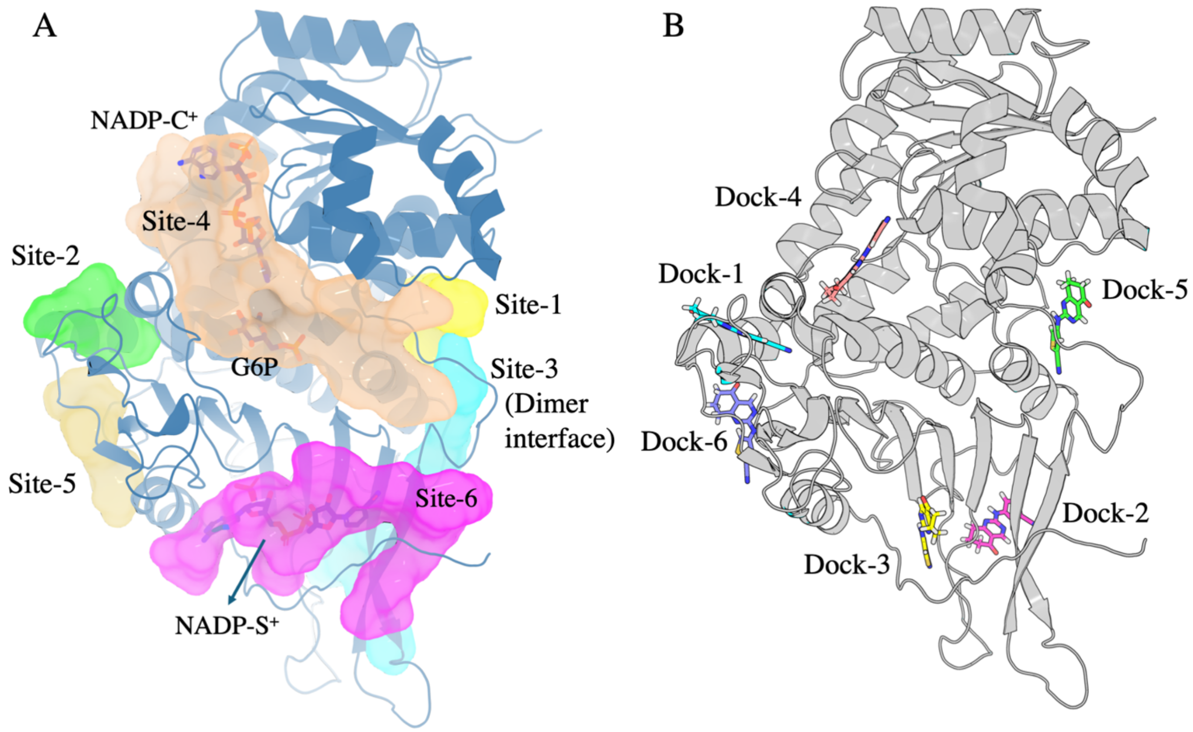

Figure S2. Identified binding pockets and top docked poses of G6PDi-1 on G6PD. (A) Binding pockets identified by SiteMap (Site-1 to Site-6) mapped onto the G6PD monomer crystal structure. The protein is shown in cartoon representation, with SiteMap cavities rendered as coloured surfaces. The catalytic $NADP^+$ (NADP-C), structural $NADP^+$ (NADP-S), and the glucose-6-phosphate (G6P) substrate are labelled for reference. The dimer interface region is highlighted. Site-5 corresponds to a distal pocket located on the opposite side of the protein relative to the dimer interface. (B) Top docked poses of G6PDi-1 (stick representation) obtained from docking against the SiteMap identified cavities. Docked poses (Dock-1 to Dock-6) are ranked according to their MM-GBSA binding energies. The association between docked poses and binding sites is reported in Table S2. These docked complexes were used as starting structures for subsequent MD simulations.

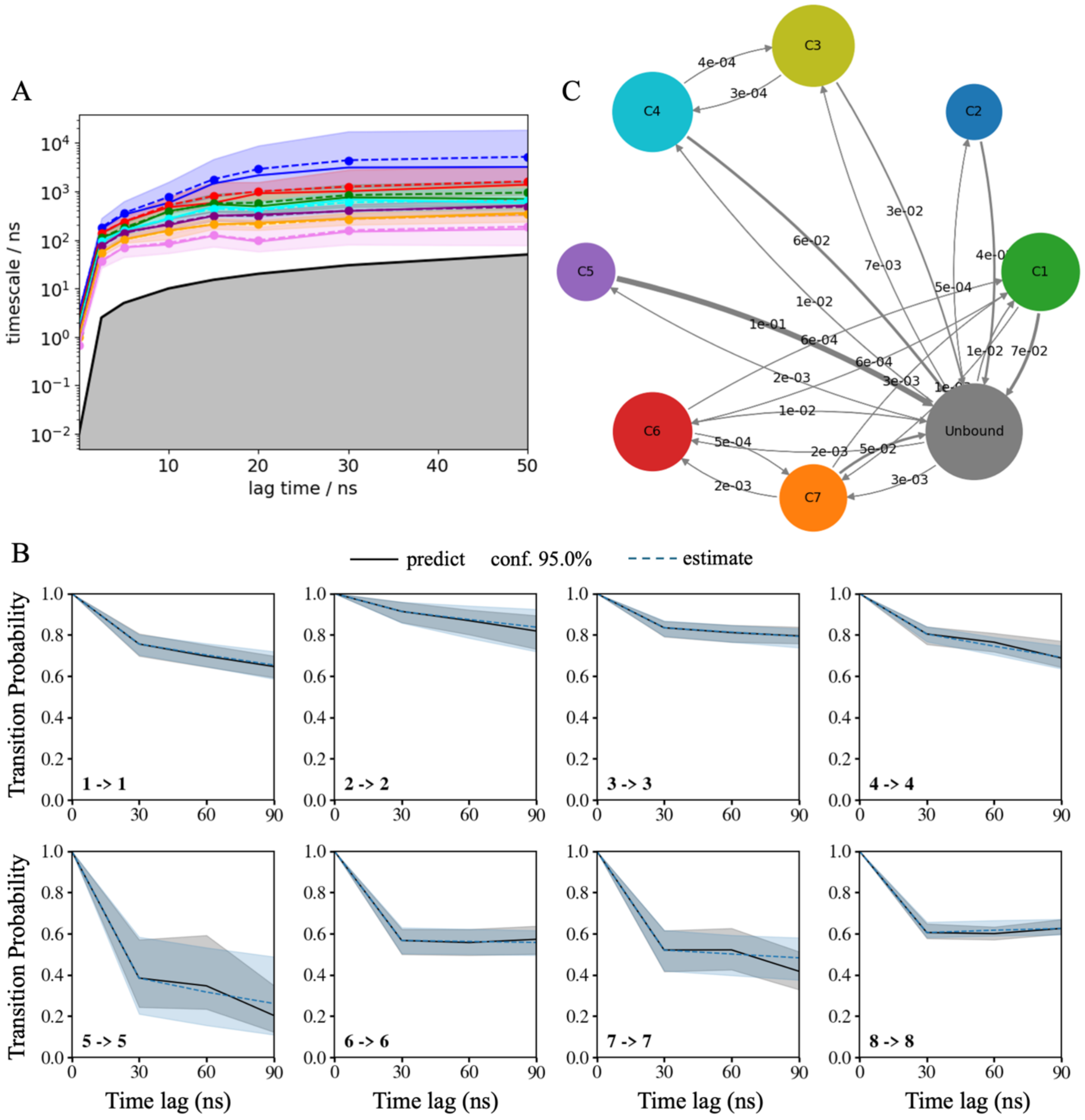

Figure S3. Hidden Markov state model (HMSM) of G6PDi-1 cavity exchange on G6PD. (A) Implied timescales as a function of lag time for the eight-state model support the choice of 30 ns lag time; shaded regions denote uncertainties. (B) Chapman-Kolmogorov (CK) validation of the eight-state HMSM. Predicted transition probabilities from the HMSM are compared with direct estimates from the simulation data at multiples of the 30 ns model lag time. (C) Kinetic network connecting the seven cavity-bound states (C1-C7) and the unbound state, where node size represents stationary population and edge thickness reflects transition probability at the chosen lag time, highlighting the unbound ensemble as the central hub for ligand exchange.

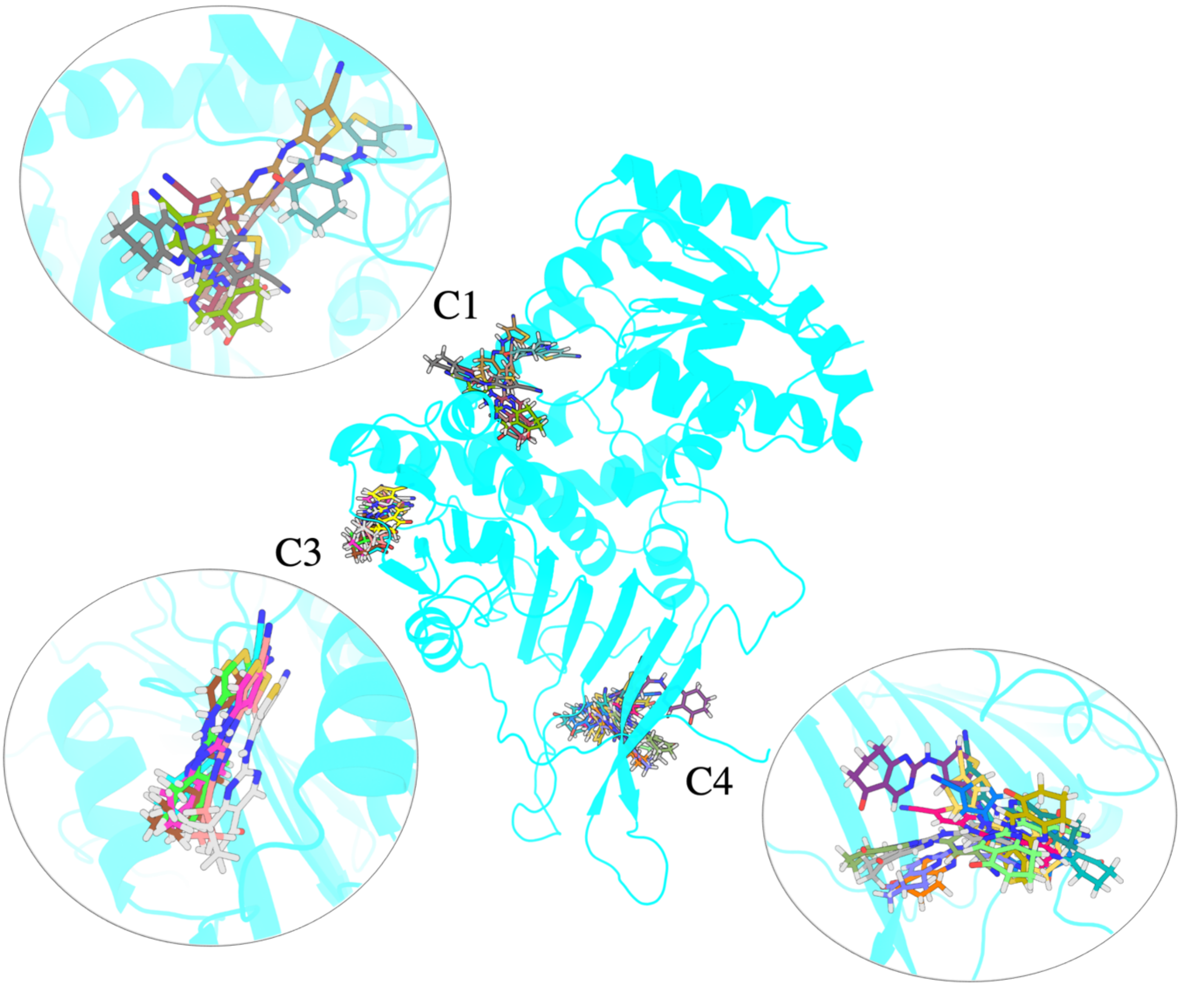

Figure S4. Representative conformations of G6PDi-1 at the binding cavities. G6PD monomer is shown in cartoon and representative ligand poses from the most populated clusters are shown as sticks at the C1, C3, and C4 sites. Clustering was performed on the protein backbone using ligand bound frames from the MD trajectories. Insets show enlarged images of each binding region. The ligand samples multiple conformations at C1 and C4, consistent with the more solvent exposed and flexible pockets, whereas C3 shows a rigid distribution of poses, indicating a more confined cavity.

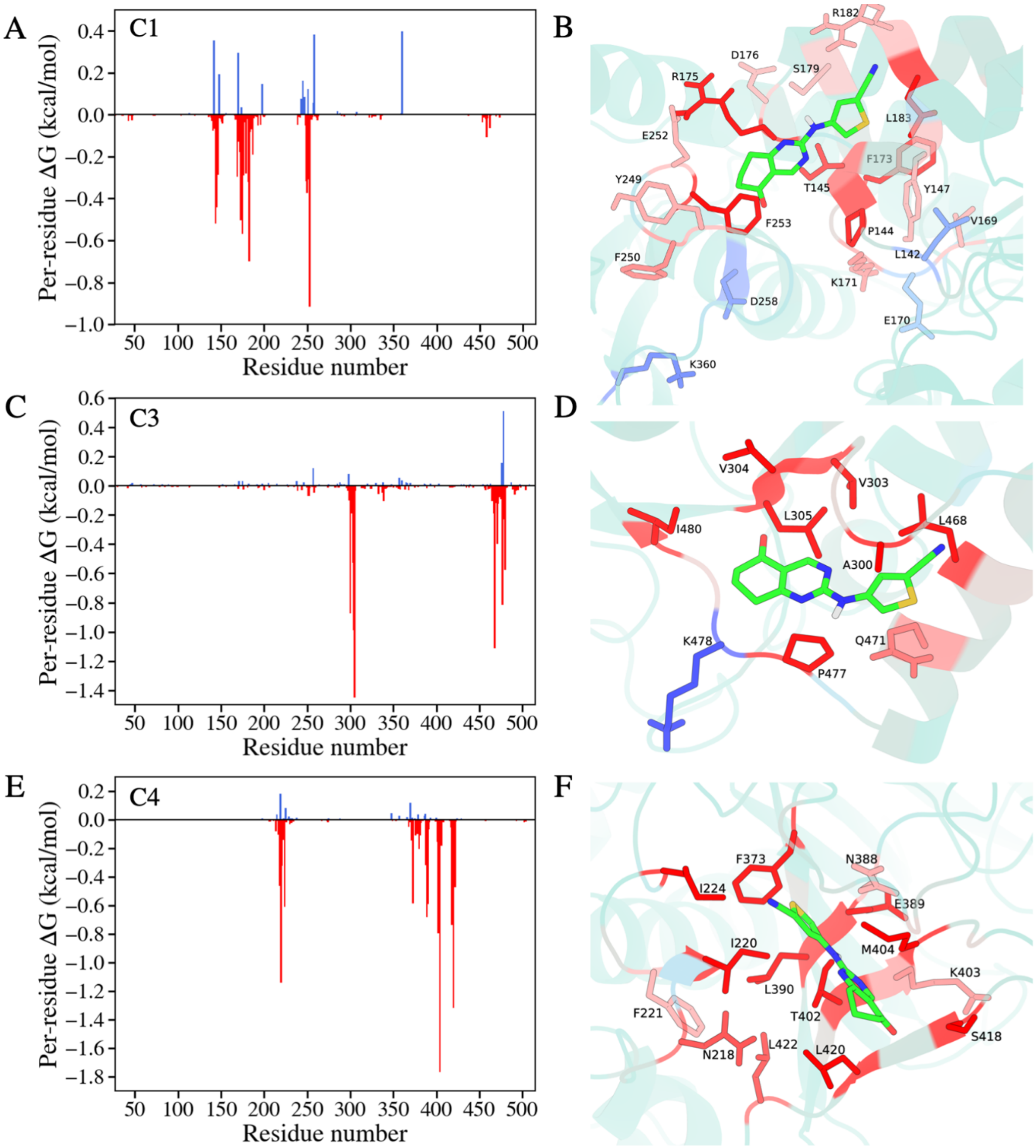

Figure S5. Residue-wise decomposition of the MM-PBSA binding free energy for G6PDi-1 bound at cavities C1, C3, and C4. The plots on the left show per-residue contributions for ligand bound at C1, C3, and C4, with favorable (negative) interactions shown in red and unfavorable (positive) interactions in blue. On the right, we show representative binding poses of G6PDi-1 at each cavity together with the surrounding residues, using the same color scheme as in the corresponding plots.

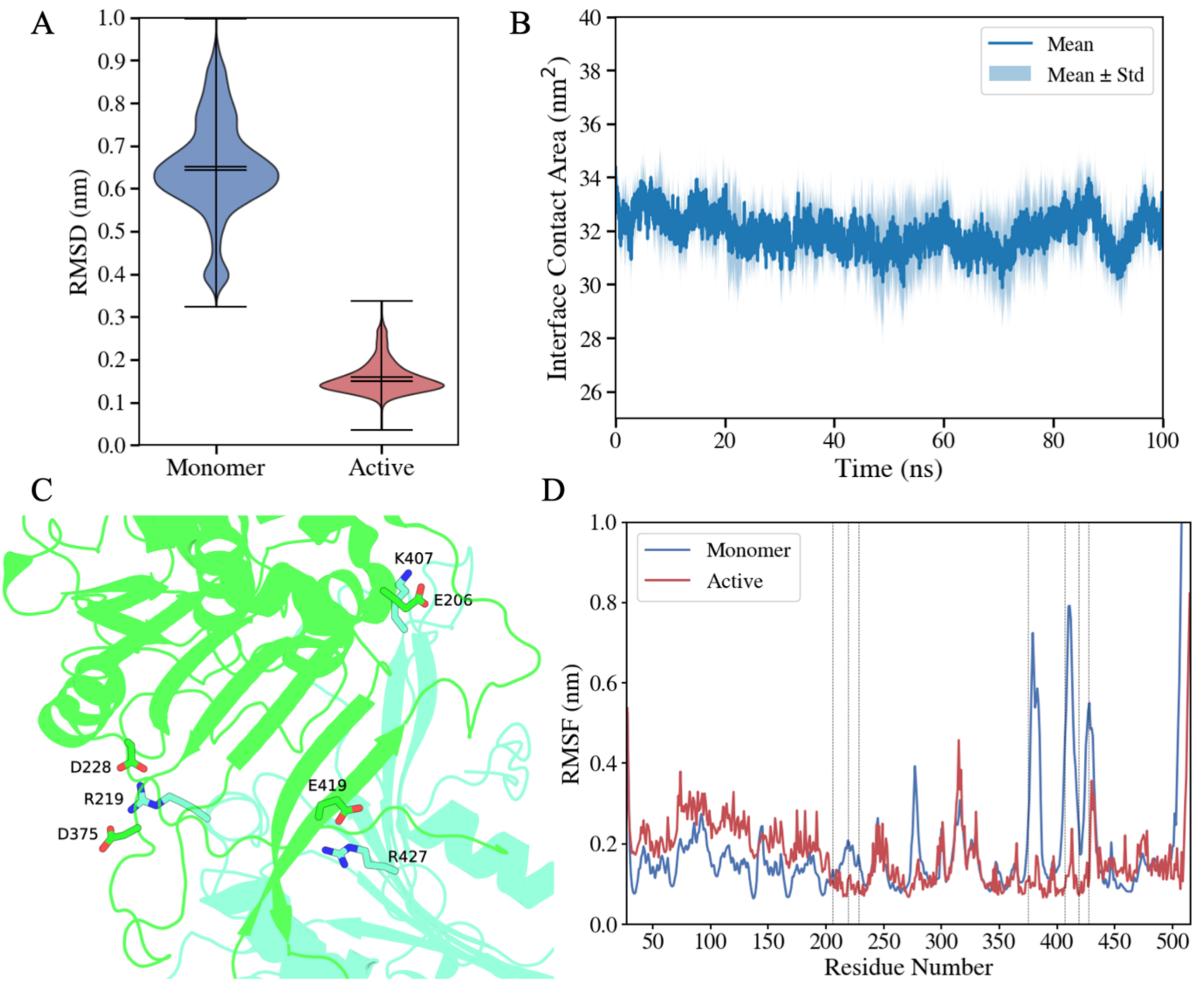

Figure S6. (A) Interface RMSD distribution for *apo* monomeric G6PD and the active oligomeric form, showing reduced structural deviation of the interface in the active state. (B) Inter-subunit contact area per monomer in the active G6PD simulations. The solid line represents the mean and the shaded region indicates the standard deviation. (C) Structural representation of the G6PD dimer, with the two subunits shown in green and cyan. Representative inter-subunit salt bridges stabilizing the interface are highlighted for one monomer. (D) Residue-wise RMSF profile of *apo* monomeric G6PD and the active form. Vertical dashed lines indicate residues involved in salt-bridge formation at the dimer interface.

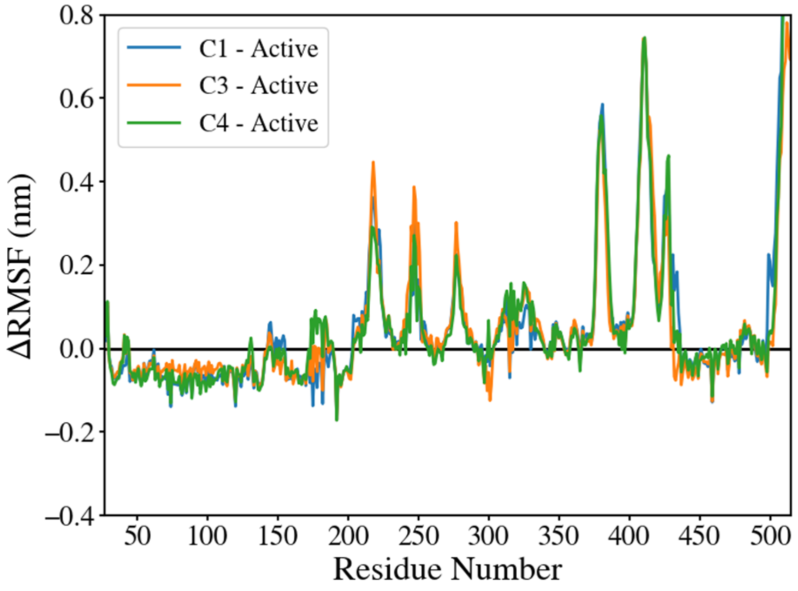

Figure S7. Difference in RMSF between the ligand bound systems (C1, C3, C4) and the active tetrameric state. Changes were calculated as $\Delta RMSF = RMSF_{Cavity} - RMSF_{Active}$ where the RMSF of a monomer in the tetrameric state was averaged over all subunits.

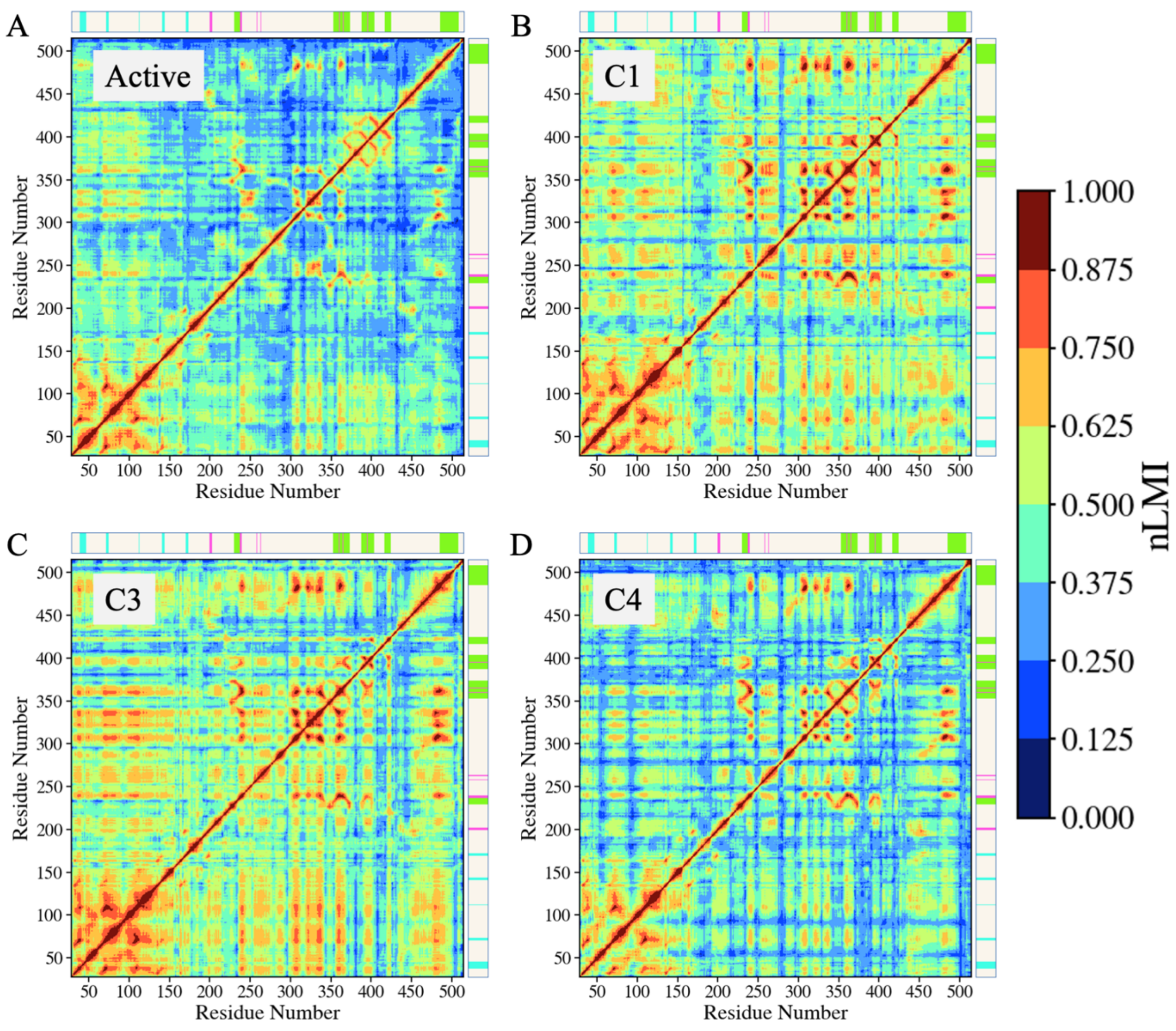

Figure S8. Residue pairwise normalized linear mutual information (nLMI) matrices calculated from MD trajectories using CorrelationPlus package for the active tetrameric state (A) and for G6PDi-1 bound at cavities C1 (B), C3 (C), and C4 (D). nLMI captures correlated motions without the angular dependence that can limit standard dynamical cross-correlation analyses. Here, in the plots, 0 value means that there is no correlation, while 1 means they are completely correlated. Compared with the active state, ligand binding at C1 and C3 produces broader off-diagonal correlation patterns, indicating increased long range coupling between residues. In contrast, the C4 bound state retains a correlation pattern closer to the active state, with more localized changes. The annotated colored dashes along the top and right sides of each nLMI map indicate residues associated with the substrate binding site (magenta), structural NADP+ site (green), and catalytic NADP+ site (cyan).

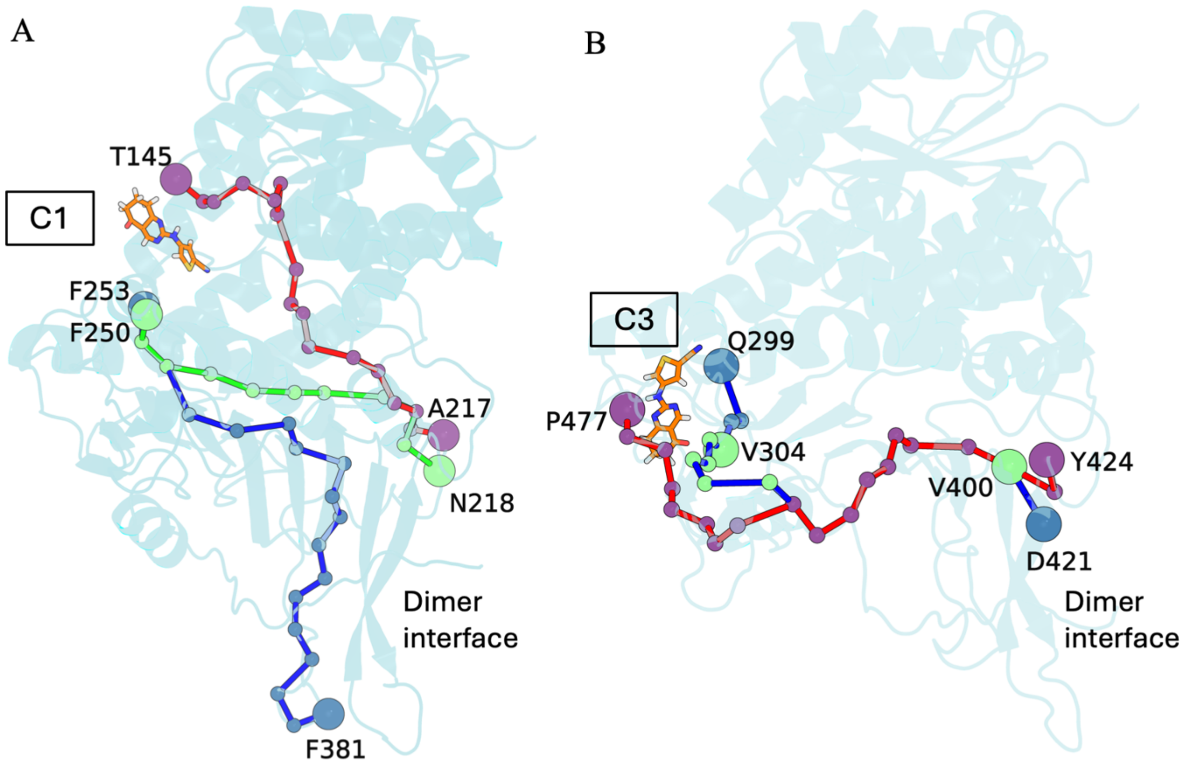

Figure S9. Structural mapping of nLMI derived allosteric pathways between ligand interacting regions and the dimer interface. Representative pathways linking G6PDi-1 interacting residues at C1 (A) and C3 (B) to dimer interface residues were mapped onto the 3D structure of G6PD. Ligand interacting residues were selected based on high contact frequency with G6PDi-1 ($>2x10^5$ contacts), while dimer-interface residues were taken from the set used in the DRMSD analysis. Residue pairs between each cavity and the dimer interface were screened using the nLMI correlation maps, retaining long-range pairs with nLMI correlation values > 0.6 and Cα distances > 20 Å. Three representative residue pairs were selected for each cavity, and the corresponding allosteric pathways were generated using the paths utility from the Correlationplus software package. The protein is shown in light cyan, and G6PDi-1 is shown in orange. Residues involved in the selected pathways are shown as spheres and connected through their Cα atoms. Different colors indicate distinct communication pathways between highly correlated residues.

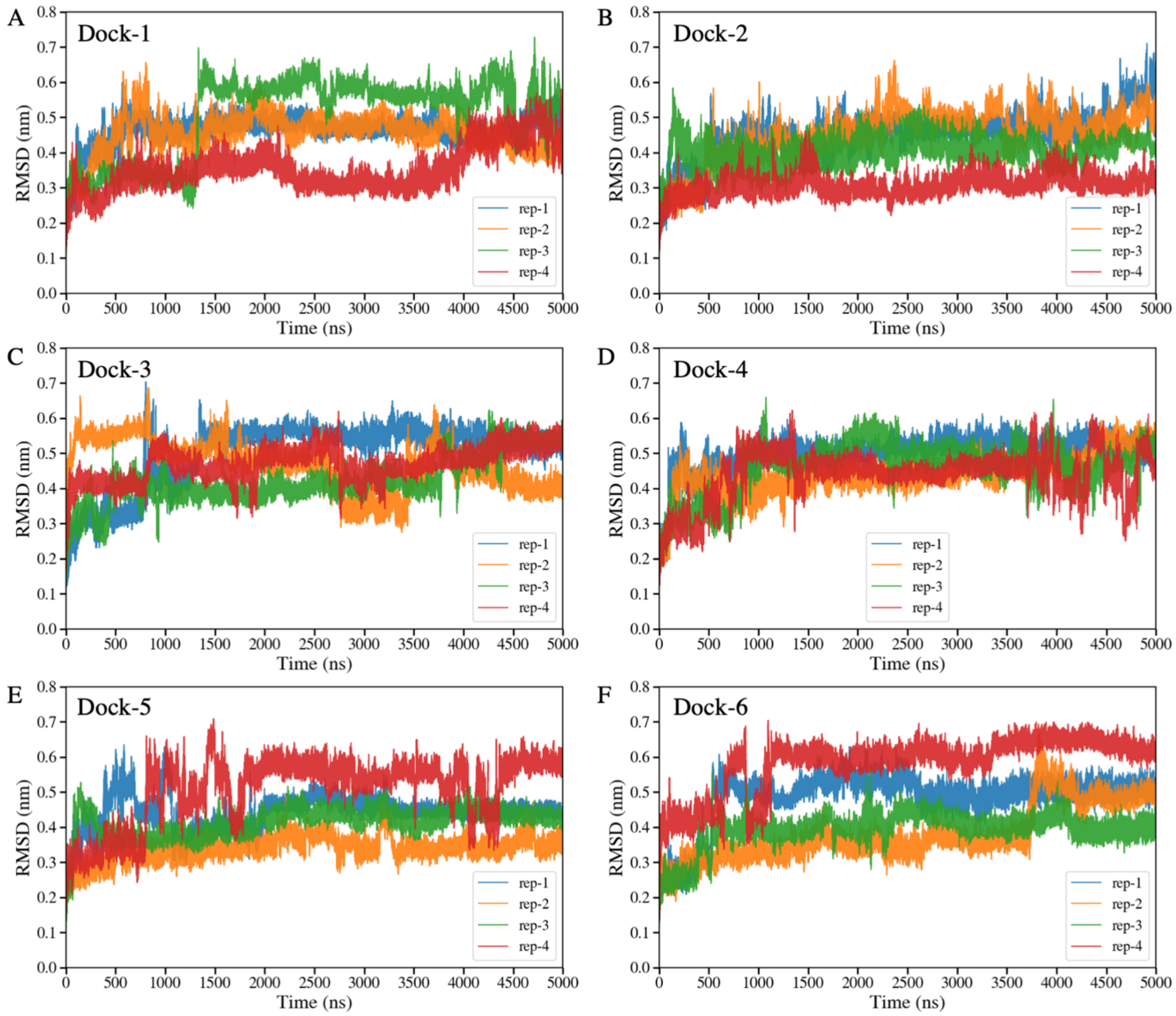

Figure S10. Root mean square deviation (RMSD) of the G6PD monomer for the six docked protein-ligand systems (Dock-1 to Dock-6) calculated over the backbone atoms. For each docked pose, four independent replicas of 5μs each were performed.

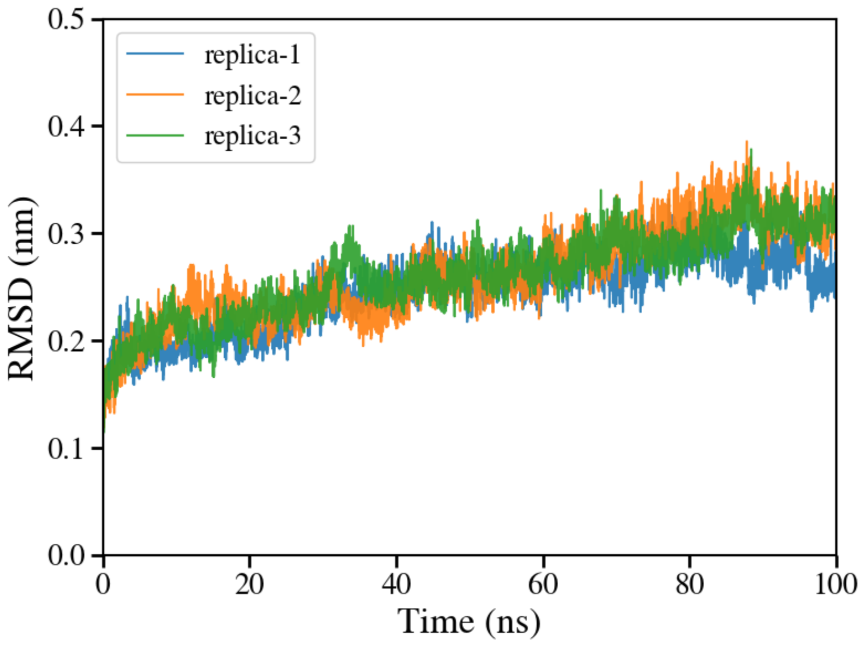

Figure S11. RMSD plot of the G6PD tetramer in active state.

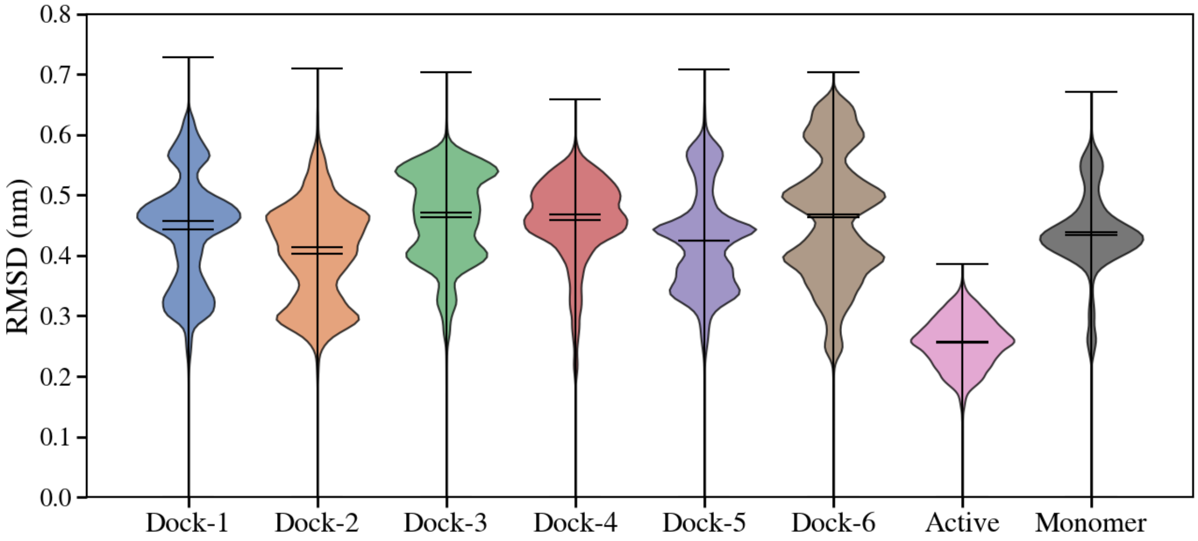

Figure S12. RMSD distributions for the six docked systems, active tetrameric state and monomeric (*apo*) state. For each docked system, RMSD values from all replicas were combined and shown as a single violin distribution. The active state and monomeric state are shown for comparison.

References

(1) Madhavi Sastry, G.; Adzhigirey, M.; Day, T.; Annabhimoju, R.; Sherman, W. Protein and Ligand Preparation: Parameters, Protocols, and Influence on Virtual Screening Enrichments. *J Comput Aided Mol Des* **2013**, *27* (3), 221–234. https://doi.org/10.1007/s10822-013-9644-8.

(2) Halgren, T. A. Identifying and Characterizing Binding Sites and Assessing Druggability. *J. Chem. Inf. Model.* **2009**, *49* (2), 377–389. https://doi.org/10.1021/ci800324m.

(3) Wei, X.; Kixmoeller, K.; Baltrusaitis, E.; Yang, X.; Marmorstein, R. Allosteric Role of a Structural NADP+ Molecule in Glucose-6-Phosphate Dehydrogenase Activity. *Proceedings of the National Academy of Sciences* **2022**, *119* (29), e2119695119. https://doi.org/10.1073/pnas.2119695119.

(4) Halgren, T. A.; Murphy, R. B.; Friesner, R. A.; Beard, H. S.; Frye, L. L.; Pollard, W. T.; Banks, J. L. Glide: A New Approach for Rapid, Accurate Docking and Scoring. 2. Enrichment Factors in Database Screening. *J. Med. Chem.* **2004**, *47* (7), 1750–1759. https://doi.org/10.1021/jm030644s.

(5) Li, J.; Abel, R.; Zhu, K.; Cao, Y.; Zhao, S.; Friesner, R. A. The VSGB 2.0 Model: A next Generation Energy Model for High Resolution Protein Structure Modeling. *Proteins: Structure, Function, and Bioinformatics* **2011**, *79* (10), 2794–2812. https://doi.org/10.1002/prot.23106.

(6) Lu, C.; Wu, C.; Ghoreishi, D.; Chen, W.; Wang, L.; Damm, W.; Ross, G. A.; Dahlgren, M. K.; Russell, E.; Von Bargen, C. D.; Abel, R.; Friesner, R. A.; Harder, E. D. OPLS4: Improving Force Field Accuracy on Challenging Regimes of Chemical Space. *J. Chem. Theory Comput.* **2021**, *17* (7), 4291–4300. https://doi.org/10.1021/acs.jctc.1c00302.

(7) Wang, E.; Sun, H.; Wang, J.; Wang, Z.; Liu, H.; Zhang, J. Z. H.; Hou, T. End-Point Binding Free Energy Calculation with MM/PBSA and MM/GBSA: Strategies and Applications in Drug Design. *Chem. Rev.* **2019**, *119* (16), 9478–9508. https://doi.org/10.1021/acs.chemrev.9b00055.

(8) Frisch, M. J.; Trucks, G. W.; Schlegel, H. B.; Scuseria, G. E.; Robb, M. A.; Cheeseman, J. R.; Scalmani, G.; Barone, V.; Petersson, G. A.; Nakatsuji, H.; Li, X.; Caricato, M.; Marenich, A. V.; Bloino, J.; Janesko, B. G.; Gomperts, R.; Mennucci, B.; Hratchian, H. P.; Ortiz, J. V.; Izmaylov, A. F.; Sonnenberg, J. L.; Williams; Ding, F.; Lipparini, F.; Egidi, F.; Goings, J.; Peng, B.; Petrone, A.; Henderson, T.; Ranasinghe, D.; Zakrzewski, V. G.; Gao, J.; Rega, N.; Zheng, G.; Liang, W.; Hada, M.; Ehara, M.; Toyota, K.; Fukuda, R.; Hasegawa, J.; Ishida, M.; Nakajima, T.; Honda, Y.; Kitao, O.; Nakai, H.; Vreven, T.; Throssell, K.; Montgomery Jr., J. A.; Peralta, J. E.; Ogliaro, F.; Bearpark, M. J.; Heyd, J. J.; Brothers, E. N.; Kudin, K. N.; Staroverov, V. N.; Keith, T. A.; Kobayashi, R.; Normand, J.; Raghavachari, K.; Rendell, A. P.; Burant, J. C.; Iyengar, S. S.; Tomasi, J.; Cossi, M.; Millam, J. M.; Klene, M.; Adamo, C.; Cammi, R.; Ochterski, J. W.; Martin, R. L.; Morokuma, K.; Farkas, O.; Foresman, J. B.; Fox, D. J. Gaussian 16 Rev. C.01, 2016.

(9) Case, D. A.; Aktulga, H. M.; Belfon, K.; Cerutti, D. S.; Cisneros, G. A.; Cruzeiro, V. W. D.; Forouzesh, N.; Giese, T. J.; Götz, A. W.; Gohlke, H.; Izadi, S.; Kasavajhala, K.; Kaymak, M. C.; King, E.; Kurtzman, T.; Lee, T.-S.; Li, P.; Liu, J.; Luchko, T.; Luo, R.; Manathunga, M.; Machado, M. R.; Nguyen, H. M.; O'Hearn, K. A.; Onufriev, A. V.; Pan, F.; Pantano, S.; Qi, R.; Rahnamoun, A.; Risheh, A.; Schott-Verdugo, S.; Shajan, A.; Swails, J.; Wang, J.; Wei, H.; Wu, X.; Wu, Y.; Zhang, S.; Zhao, S.; Zhu, Q.; Cheatham, T. E. I.; Roe, D. R.; Roitberg, A.; Simmerling, C.; York, D. M.; Nagan, M. C.; Merz, K. M. Jr. AmberTools. *J. Chem. Inf. Model.* **2023**, *63* (20), 6183–6191. https://doi.org/10.1021/acs.jcim.3c01153.

(10) Case, D. A.; Cerutti, D. S.; Cruzeiro, V. W. D.; Darden, T. A.; Duke, R. E.; Ghazimirsaeed, M.; Giambaşu, G. M.; Giese, T. J.; Götz, A. W.; Harris, J. A.; Kasavajhala, K.; Lee, T.-S.; Li, Z.; Lin, C.; Liu, J.; Miao, Y.; Salomon-Ferrrer, R.; Shen, J.; Snyder, R.; Swails, J.; Walker, R. C.; Wang, J.; Wu, X.; Zeng, J.; Cheatham III, T. E.; Roe, D. R.; Roitberg, A.; Simmerling, C.; York, D. M.; Nagan, M. C.; Merz, K. M. Jr. Recent Developments in Amber Biomolecular Simulations. *J. Chem. Inf. Model.* **2025**, *65* (15), 7835–7843. https://doi.org/10.1021/acs.jcim.5c01063.

(11) Wang, J.; Wolf, R. M.; Caldwell, J. W.; Kollman, P. A.; Case, D. A. Development and Testing of a General Amber Force Field. *Journal of Computational Chemistry* **2004**, *25* (9), 1157–1174. https://doi.org/10.1002/jcc.20035.

(12) Stewart, J. J. P. Optimization of Parameters for Semiempirical Methods VI: More Modifications to the NDDO Approximations and Re-Optimization of Parameters. *J Mol Model* **2013**, *19* (1), 1–32. https://doi.org/10.1007/s00894-012-1667-x.

(13) Stephens, P. J.; Devlin, F. J.; Chabalowski, C. F.; Frisch, M. J. Ab Initio Calculation of Vibrational Absorption and Circular Dichroism Spectra Using Density Functional Force Fields. *J. Phys. Chem.* **1994**, *98* (45), 11623–11627. https://doi.org/10.1021/j100096a001.

(14) Weigend, F.; Ahlrichs, R. Balanced Basis Sets of Split Valence, Triple Zeta Valence and Quadruple Zeta Valence Quality for H to Rn: Design and Assessment of Accuracy. *Phys. Chem. Chem. Phys.* **2005**, *7* (18), 3297–3305. https://doi.org/10.1039/B508541A.

(15) Grimme, S.; Antony, J.; Ehrlich, S.; Krieg, H. A Consistent and Accurate Ab Initio Parametrization of Density Functional Dispersion Correction (DFT-D) for the 94 Elements H-Pu. *J. Chem. Phys.* **2010**, *132* (15), 154104. https://doi.org/10.1063/1.3382344.

(16) Bayly, C. I.; Cieplak, P.; Cornell, W.; Kollman, P. A. A Well-Behaved Electrostatic Potential Based Method Using Charge Restraints for Deriving Atomic Charges: The RESP Model. *J. Phys. Chem.* **1993**, *97* (40), 10269–10280. https://doi.org/10.1021/j100142a004.

(17) Besler, B. H.; Merz Jr., K. M.; Kollman, P. A. Atomic Charges Derived from Semiempirical Methods. *Journal of Computational Chemistry* **1990**, *11* (4), 431–439. https://doi.org/10.1002/jcc.540110404.

(18) Shirts, M. R.; Klein, C.; Swails, J. M.; Yin, J.; Gilson, M. K.; Mobley, D. L.; Case, D. A.; Zhong, E. D. Lessons Learned from Comparing Molecular Dynamics Engines on the SAMPL5 Dataset. *J Comput Aided Mol Des* **2017**, *31* (1), 147–161. https://doi.org/10.1007/s10822-016-9977-1.

(19) Abraham, M. J.; Murtola, T.; Schulz, R.; Páll, S.; Smith, J. C.; Hess, B.; Lindahl, E. GROMACS: High Performance Molecular Simulations through Multi-Level Parallelism from Laptops to Supercomputers. *SoftwareX* **2015**, *1–2*, 19–25. https://doi.org/10.1016/j.softx.2015.06.001.

(20) Tian, C.; Kasavajhala, K.; Belfon, K. A. A.; Raguette, L.; Huang, H.; Migues, A. N.; Bickel, J.; Wang, Y.; Pincay, J.; Wu, Q.; Simmerling, C. ff19SB: Amino-Acid-Specific Protein Backbone Parameters Trained against Quantum Mechanics Energy Surfaces in Solution. *J. Chem. Theory Comput.* **2020**, *16* (1), 528–552. https://doi.org/10.1021/acs.jctc.9b00591.

(21) Jorgensen, W. L.; Chandrasekhar, J.; Madura, J. D.; Impey, R. W.; Klein, M. L. Comparison of Simple Potential Functions for Simulating Liquid Water. *J. Chem. Phys.* **1983**, *79* (2), 926–935. https://doi.org/10.1063/1.445869.

(22) Darden, T.; York, D.; Pedersen, L. Particle Mesh Ewald: An N·log(N) Method for Ewald Sums in Large Systems. *J. Chem. Phys.* **1993**, *98* (12), 10089–10092. https://doi.org/10.1063/1.464397.

(23) Hess, B.; Bekker, H.; Berendsen, H. J. C.; Fraaije, J. G. E. M. LINCS: A Linear Constraint Solver for Molecular Simulations. *Journal of Computational Chemistry* **1997**, *18* (12), 1463–1472. https://doi.org/10.1002/(SICI)1096-987X(199709)18:12%253C1463::AID-JCC4%253E3.0.CO;2-H.
(24) Bussi, G.; Donadio, D.; Parrinello, M. Canonical Sampling through Velocity Rescaling. *J. Chem. Phys.* **2007**, *126* (1). https://doi.org/10.1063/1.2408420.
(25) Bernetti, M.; Bussi, G. Pressure Control Using Stochastic Cell Rescaling. *J. Chem. Phys.* **2020**, *153* (11). https://doi.org/10.1063/5.0020514.
(26) Huang, J.; Rauscher, S.; Nawrocki, G.; Ran, T.; Feig, M.; de Groot, B. L.; Grubmüller, H.; MacKerell, A. D. CHARMM36m: An Improved Force Field for Folded and Intrinsically Disordered Proteins. *Nat Methods* **2017**, *14* (1), 71–73. https://doi.org/10.1038/nmeth.4067.
(27) Vanommeslaeghe, K.; MacKerell, A. D. Jr. Automation of the CHARMM General Force Field (CGenFF) I: Bond Perception and Atom Typing. *J. Chem. Inf. Model.* **2012**, *52* (12), 3144–3154. https://doi.org/10.1021/ci300363c.
(28) Noé, F.; Wu, H.; Prinz, J.-H.; Plattner, N. Projected and Hidden Markov Models for Calculating Kinetics and Metastable States of Complex Molecules. *J. Chem. Phys.* **2013**, *139* (18), 184114. https://doi.org/10.1063/1.4828816.
(29) Scherer, M. K.; Trendelkamp-Schroer, B.; Paul, F.; Pérez-Hernández, G.; Hoffmann, M.; Plattner, N.; Wehmeyer, C.; Prinz, J.-H.; Noé, F. PyEMMA 2: A Software Package for Estimation, Validation, and Analysis of Markov Models. *J. Chem. Theory Comput.* **2015**, *11* (11), 5525–5542. https://doi.org/10.1021/acs.jctc.5b00743.
